# ‘This country is OURS’: The exclusionary potential of collective psychological ownership

**DOI:** 10.1111/bjso.12386

**Published:** 2020-06-07

**Authors:** Tom Nijs, Borja Martinovic, Maykel Verkuyten, Constantine Sedikides

**Affiliations:** ^1^ Ercomer Utrecht University The Netherlands; ^2^ Centre for Research on Self and Identity University of Southampton UK

**Keywords:** collective psychological ownership, anti‐immigrant attitudes, anti‐EU attitudes, Brexit referendum, exclusive determination right

## Abstract

Political campaign slogans, such as ‘Take back control of our country’ (United Kingdom Independence Party) and ‘The Netherlands ours again’ (Dutch Party for Freedom), indicate that right‐wing populism appeals to the belief that the country is ‘ours’, and therefore, ‘we’ have the exclusive right to determine what happens. We examined this sense of ownership of the country (i.e. collective psychological ownership [CPO]) with the related determination right in relation to exclusionary attitudes and voting behaviour. Among Dutch (Study 1, *N* = 572) and British (Study 2, *N* = 495) participants, we found that CPO explained anti‐immigrant and anti‐EU attitudes, and these attitudes in turn accounted for voting ‘leave’ in the 2016 Brexit referendum in the British sample (Study 2). Additionally, CPO was more strongly related to negative immigrant attitudes among right‐wing Dutch participants, whereas it was more strongly related to negative EU attitudes and voting ‘leave’ among left‐wing British participants. CPO contributes to the understanding of critical contemporary social attitudes and political behaviour.

Through such political campaign slogans as ‘Take back control of our country’ and ‘The Netherlands ours again’, right‐wing populist parties (United Kingdom Independence Party and Dutch Party for Freedom, respectively) endeavour to appeal to beliefs that the country is ‘ours’ and therefore ‘we’ are its rightful owners. These political parties appeal to people's sense of ownership and the (arguably) related exclusive determination right to back up opposition to immigration and European integration (Partij Voor de Vrijheid, 2012; Vlaams Belang, 2019). The United Kingdom Independence Party also used ownership rhetoric in European Union (EU) ‘leave’ campaigns (Cap, 2017; Portice & Reicher, 2018). Indeed, people may feel not only that objects, places, or ideas are ‘theirs’, but also that their ingroup owns a complex entity such as a country (Brylka, Mähönen, & Jasinskaja‐Lahti, 2015; Verkuyten & Martinovic, 2017). This ingroup perception, labelled *collective psychological ownership* (CPO; Pierce & Jussila, 2009; Verkuyten & Martinovic, 2017), implies a right to control what is ‘ours’ – *exclusive determination right* – and can contribute to the strong ‘us‐them’ distinction that is characteristic of right‐wing populism. We examined whether CPO implies an exclusive determination right that accounts for anti‐immigration and anti‐EU attitudes of the Dutch (Study 1) and the British (Study 2), as well as whether these attitudes, in turn, explain voting behaviour in the 2016 Brexit referendum (Study 2). We additionally examined whether a sense of collective ownership is especially related to exclusionary attitudes and behaviour among politically right‐wing people.

## Collective psychological ownership and exclusive determination right

Psychological ownership implies the subjective sense of control and power over things. It is being tethered to the object, place, or idea perceived to be one's own, even if one does not own something in legal terms (Gregg, Mahadevan, & Sedikides, 2017; Pierce, Kostova, & Dirks, 2001). The sense of ownership has its foundations in the psychology of possessions (Rochat, 2014), develops very early in life, and probably has roots in evolutionary history, as is illustrated in the territorial instinct that is found in many species (Hinde, 1970). Children as young as two understand that something is ‘mine’ and not ‘yours’ (Ross, Friedman, & Field, 2015; Rossano, Rakoczy, & Tomasello, 2011), and 3‐year‐olds recognize the person who controls the use of an object as the owner (Neary, Friedman, & Burnstein, 2009; Shaw, Li, & Olson, 2012).

People not only experience personal psychological ownership, but can also perceive something to be owned by their group. When people have a sense of ‘us’, they can also have a sense of ‘ours’, referred to as CPO. Organizational scholars have argued that team members in an organization can perceive their team to have collective ownership of their work, their working space, and their work outcomes (Pierce & Jussila, 2009; Pierce, Jussila, & Li, 2017).^1^ These perceptions relate to the question of ‘what we control’, which differs from questions of group identity (‘who we are’) and group resources (‘what we need’) (Verkuyten & Martinovic, 2017). Crucially, people can also perceive collective ownership of a country (Brylka *et al*., 2015; Verkuyten & Martinovic, 2017). Although legal regulations about historical sovereignty rights often serve as a basis for such ownership claims (Gans, 2001), perceptions of ‘our’ country can exist independently of legal regulations. These perceptions are expected to be relatively stable individual dispositions, as some individuals have stronger general tendencies to experience ownership than others (Pierce, Kostova, & Dirks, 2003).

Given that ownership rhetoric is frequently implemented by right‐wing populist politicians, CPO may help to explain the attractiveness of right‐wing populist messages. Right‐wing populism is an ideology defined by a (1) vertical ‘us‐them’ distinction between ‘the pure people’ and ‘the corrupt elite’ (Mudde, 2007) and (2) horizontal ‘us‐them’ distinction in which ‘the pure people’ are distinguished from immigrant and ethnic minority groups, sometimes labelled ‘the dangerous others’ (Albertazzi & McDonnell, 2007; Brubaker, 2019). Right‐wing populism has appropriated these distinctions as a basis of morality, but also as a basis of entitlement. ‘The people’ are not only distinct from ‘the corrupt elite’ or ‘the dangerous others’ because they are morally good, but also because they are entitled to be ‘masters in their own homes’ (Albertazzi & McDonnell, 2007, p. 6).

Collective psychological ownership legitimizes the populist ‘us‐them’ distinction, as it implies specific rights. Ownership confers rights and privileges with respect to that which is owned and thereby determines the entitlements of owners in relation to non‐owners. Philosophers have argued that ownership is accompanied by the right to use one's property, transfer it to others, and exclude others from using it (Snare, 1972). The latter is considered the defining feature of ownership (Merrill, 1998), and so we focus on it. We conceptualize *exclusive determination right* as an owner's right to determine what happens with the ‘property’, and hence to exclude non‐owners (Katz, 2008). The idea that ‘the people’ have the exclusive right to determine the fate of the nation lies at the heart of populism (Akkerman, Mudde, & Zaslove, 2013; Mudde, 2010). This right affords a sense of power and control, which is part of the psychology of possession and a central motive behind the endorsement of CPO (Rochat, 2014; Verkuyten & Martinovic, 2017). Given that ownership contains not only the exclusive determination right, but also other rights (right to use and transfer; Snare, 1972), we distinguish conceptually and empirically between CPO and exclusive determination right, while acknowledging the centrality of the exclusive determination right for CPO.

## Ownership and attitudes towards immigrant minorities and European integration

Populist right‐wing politicians often refer explicitly to CPO and the exclusive determination right when combining their opposition to two key issues, immigration and European integration (Lubbers & Coenders, 2017; Mudde, 2007). This point is illustrated by a quote from a speech given by the leader of the Dutch Party for Freedom: ‘When leaving the EU and Eurozone we will be in charge of our own rules again, like about who enters our country, immigration, and our own currency’ (Wilders, 2012). Given that CPO is often based on arguments of autochthony (‘we were here first’) and investment (‘we built this country’) (Verkuyten, Sierksma, & Thijs, 2015), right‐wing populism may not consider newcomers as rightful owners of the country. Therefore, this ideology may not regard the exclusion of immigrant minorities as unjust or discriminatory, but rather as a self‐evident right that accompanies CPO (Merrill, 1998; Verkuyten & Martinovic, 2017). CPO can be used to define group‐based hierarchies without raising moral questions, because ownership involves a consensually shared understanding about how to determine entitlements (Costa‐Lopes, Dovidio, Pereira, & Jost, 2013). General senses of both personal and group entitlement are related to more negative outgroup attitudes, as they imply acceptable differences between individuals and groups (Anastasio & Rose, 2014; Blumer, 1958). Right‐wing populists, then, may use the rhetoric of CPO and its exclusive determination right as a basis for opposing immigrants (Fine, 2013).

Collective psychological ownership and its exclusive determination right may also be associated with opposition to European integration. Involvement of the EU in what are perceived to be national matters may be regarded as international elite interfering with the exclusive right to make decisions about one's own country, which taps into the vertical ‘people‐elite’ distinction of right‐wing populism (Føllesdal, 1998). European integration has led to common policies in a range of domains, such as security (Europol) and monetary (the Euro; European Parliament, 2014) all of which can be seen as examples of ‘interference’ by the EU elite not listening to the people (Harmsen, 2010). In particular, the Schengen Agreement that assured the free movement of European citizens across European nation states (Baldoni, 2003), and increasingly centralized immigration and asylum policies concerning immigration from outside the EU (Hatton, 2015), may, from the perspective of ‘our’ country, be regarded as incompatible with ‘our’ right to determine about the entry of newcomers.

Consequently, we hypothesize that CPO is associated with more negative attitudes towards immigrant minorities (H1a) and European integration (H1b) and that the perceived exclusive determination right mediates these associations (H2a and H2b).

## Pro‐Brexit vote

A key element of ownership is establishing, communicating, and maintaining what is owned (Brown, Lawrence, & Robinson, 2005). To do so, it is necessary to exclude others and take action when the exclusivity of one's rights is not guaranteed. Voting is such an action.

The 2016 Brexit referendum was a political event that may have been influenced by ownership concerns. On 23 June 2016, 51.9% of the British electorate voted to leave the EU. Many voters and politicians perceived the referendum as an opportunity to regain control over what is ‘ours’ (Andreouli & Nicholson, 2018; Capelos & Katsanidou, 2018; Hobolt, 2016; Portice & Reicher, 2018). Controlling national legislation and borders were the most salient themes among Leave supporters (Andreouli & Nicholson, 2018), and the United Kingdom Independence Party campaigned for ‘leave’ with the characteristic slogan ‘Take back control of our country’.

We proceeded to examine whether the negative attitudinal consequences of ownership translate into a pro‐Brexit vote, thereby focusing on behaviour. Concerns about immigrants' negative impact on the British economy, culture, and welfare state were drivers of the pro‐Brexit vote (Goodwin & Milazzo, 2017; Hutchings & Sullivan, 2019). The vote is further explained by negative attitudes towards European integration, and specifically by cost and benefit concerns of the integration for employment, welfare, and freedom of movement (Vasilopoulou, 2016). As such, we expect CPO to be associated with a higher likelihood of pro‐Brexit voting (H3) via exclusive determination right (H3a) and, in turn, negative attitudes towards immigrant minorities (H3b) and European integration (H3c).

Although it is possible that CPO is used to justify one's pre‐existing negative attitudes towards immigrants and the EU or Brexit voting, we argue that CPO influences these attitudes and voting behaviour. CPO is a general underlying belief about what is ‘ours’ that translates into more specific attitudes varying in ideological relevance across context and time (Verkuyten & Martinovic, 2017). This is similar to ideological beliefs influencing specific attitudes (Jost, 2006) and nationalism driving outgroup attitudes (Wagner, Becker, Christ, Pettigrew, & Schmidt, 2010). Moreover, based on voting behaviour theory, we posit that people cast their votes motivated by their beliefs and attitudes (Campbell, Converse, Miller, & Stokes, 1980; Steenbergen, 2010), and a sense of group threat drives rather than results from right‐wing populist voting (Berning & Schlueter, 2016). Admittedly, though, our research designs prevent conclusions about causality, and so we cannot rule out the possibility of bidirectional associations.

## Political ideology

Although a substantial portion of the electorate might concur with populist politicians' slogans that the country is ‘ours’ and therefore ‘we’ have exclusive determination rights, not all people will consent with exclusionary attitudes and behaviour. Such consent may be primarily found among right‐wing individuals. According to the motivated social cognition model (Jost, Glaser, Kruglanski, & Sulloway, 2003), left‐wing and right‐wing individuals are distinguished in terms of their attitudes on two domains: tradition (vs. change) and equality (vs. dominance). People on the political right endorse traditionalism and conformity, while justifying inequalities between individuals and groups. In contrast, a left‐wing orientation is associated with openness to experiences as well as preferences for greater equality and diminishing group dominance (Jost, 2006). Given that right‐wing individuals generally have fewer problems with inequalities and value the status quo, they will likely translate endorsement of ownership and its exclusive determination right into exclusionary reactions (Mudde, 2007). Hence, we will use the exclusive determination right as a basis for exclusionary attitudes and behaviour. We hypothesize that the exclusive determination right is especially related to more negative attitudes towards immigrant minorities and European integration among right‐wing individuals (H4).

## Potential confounds

We further examined whether these negative attitudes (and downstream consequences) are explained by CPO above‐and‐beyond other relevant constructs. The exclusive determination right affords a sense of control, which is the primary need fulfilled by ownership (Beggan, 1991; Furby, 1978). However, ownership can additionally furnish a sense of identity and belongingness (Pierce *et al*., 2001; Porteous, 1976), and indeed, CPO is related but relatively independent from national identification and place attachment (Brylka *et al*., 2015; Storz *et al*., 2020). Furthermore, national identification is a constituent aspect of right‐wing populism (Brubaker, 2019; Lubbers, 2019) that is linked to negative attitudes towards immigrants (Pehrson, Vignoles, & Brown, 2009), the EU (Carey, 2002), and the pro‐Brexit vote (Hobolt, 2016). Place attachment entails a positive affective bond between an individual and a specific territory (Scannell & Gifford, 2010), a sense that ‘I belong to the place’, whereas CPO concerns the perception that ‘the place belongs to us’.

Exclusionary reactions can further be explained by adherence to state sovereignty. Sovereignty is a political principle that refers to the supreme authority to rule without outside interference. It was used in the Brexit debate to argue against ‘Brussels bureaucrats and elites’ making decisions about national matters, including immigration (Ringeisen‐Biardeaud, 2017). Both sovereignty and CPO can account for ‘why we get to decide’. However, whereas sovereignty is concerned with the authority in the decision‐making process of the state, CPO relates to the question whether an ‘object’ belongs to us and is ours to control (Ripstein, 2017). Based on the principle of sovereignty, people may oppose further European integration, because it impedes the possibility of national governments to decide on what is good for society. However, people may also oppose further European integration simply because they believe they themselves are entitled to control what is ‘theirs’.

We examine whether the associations between CPO and exclusionary outcomes are independent of national identification, place attachment, and adherence to sovereignty. We expected this to be the case because CPO has its basis in the psychology of possession (Rochat, 2014) and is not directly concerned with the questions of ‘who we are’, ‘where do we belong’ or ‘who decides’, but rather with the question ‘what do we control’ (Brown *et al*., 2005).

## Overview

In two studies involving Dutch (Study 1) and British (Study 2) national majority samples, we examined whether CPO is related to more negative attitudes towards immigrant minorities and European integration, via exclusive determination right. In Study 2, we additionally tested whether these exclusionary attitudes accounted for voting in favour of Brexit. We considered the moderating influence of political ideology in both studies.

The Netherlands and the United Kingdom are similar in regard to their long‐established liberal democracies, recent mass immigration, and rapid rise of populist right‐wing parties. The most relevant difference for our purposes is that the United Kingdom citizens voted for leaving the EU in the 2016 Brexit referendum. The British data offer the opportunity to test for the role of CPO in voting behaviour and the Dutch data allow to examine the role of CPO in attitudes towards European unification in a context where this topic is less hotly debated than in the British context.^2^ Although the Dutch Party for Freedom called for a Dutch EU membership referendum, Dutch mainstream parties are pro‐EU (Hobolt, 2016), and the Dutch are much less Eurosceptic than the British (Stokes, 2016), which renders ‘Nexit’ unlikely.

## STUDY 1

We tested whether CPO is related to more negative attitudes towards immigrant minorities and European integration, and whether these associations are mediated by perceived exclusive determination right. Furthermore, we considered the moderating influence of political ideology on the association between exclusive determination right and attitudes towards immigrant minorities and European integration. We controlled for national identification, place attachment, and demographic characteristics.

### Sample

We surveyed 608 participants via the Dutch online platform Thesistools (2019). Based on sample size calculator software for structural equation modelling (Soper, 2017), the main model with 20 observed indicators and three latent variables requires 323 participants to detect a medium‐sized effect (Cohen's *d* = 0.4) at the .05 alpha level. The final model including control variables was more complex, and so we aimed for a larger sample size. We excluded 27 participants who did not answer the political ideology question,^3^ and nine participants because they, or one of their parents, were not born in the Netherlands. The final sample (*N* = 572), although not representative of the Dutch majority population, was diverse in terms of sex (235 [41%] women, 335 men, 2 unreported), age (19–87, *M* = 60.17, *SD* = 13.00), and education level (11% low secondary school or less, 29% high school or vocational training, and 60% [applied] university).

### Measures

#### Collective psychological ownership

We adapted four items from a measure designed to assess CPO in organizational settings (Pierce *et al*., 2017). Participants read: ‘Think about the house, automobile, work space, or some other item that you own or co‐own with someone, and the experiences and feelings associated with the statement “THIS IS MINE/THIS IS OURS!”. The following statements refer to the feeling of being a co‐owner of a country, The Netherlands. Indicate the degree to which you personally disagree or agree with these statements’: ‘I think that this country is owned by us, the Dutch,’ ‘I feel that this country belongs to us, the Dutch’, ‘I feel that this country is collectively owned by us, the Dutch,’ ‘I feel as though we, the Dutch, own this country together’ (1 = *strongly disagree*, 7 = *strongly agree*; α = .95). Beforehand, participants were informed that, by ‘The Dutch’, we referred to people with no migration background.

#### Exclusive determination right

We asked participants to what extent they disagreed or agreed that the Dutch can claim the following rights: ‘The exclusive right to determine matters that concern The Netherlands’, ‘The exclusive right to determine the rules of the game in The Netherlands’, ‘The exclusive right to determine who will be allowed in The Netherlands’, and ‘The exclusive right to determine what happens to The Netherlands in the future’ (1 = *strongly disagree*, 7 = *strongly agree*; α = .96).

#### Immigrant minority attitudes

We used a feeling thermometer, a reliable measurement (Alwin, 1997) that correlates with subtle prejudice assessments (Dovidio, Kawakami, & Beach, 2001). It ranged from 0° (cold) to 100° (warm), with 10° increments (11‐point scale). Participants rated their feeling towards 10 immigrant minority groups in the Netherlands: Antilleans, Bulgarians, Moroccans, Poles, Surinamese, Turks, refugees, asylum seekers, people who entered the Netherlands illegally, people who overstayed their resident permits (α = .94).

#### European integration attitudes

We used an item from the European Social Survey (2018): ‘Concerning the European Union, some people think European integration should go further. Others think it has already gone too far. What describes your position best?’ (1 = *European integration has gone way too far*, 7 = *European integration should go a lot further*).

#### Political ideology

We asked participants to place themselves on a 5‐point scale (1 = *political left*, 2 = *centre left*, 3 = *middle*, 4 = *centre right*, 5 = *right*) that is a useful indicator of general political orientation (Jost, 2006).

#### National identification

We used three items (Martinovic & Verkuyten, 2012): ‘I strongly feel Dutch’, ‘Being Dutch is important to me’, ‘I identify with other Dutch people’ (1 = *strongly disagree*, 7 = *strongly agree*; α = .87).

#### Place attachment

We used three items that we adjusted from measures of attachment to one's neighbourhood (Hernández, Carmen Hidalgo, Salazar‐Laplace, & Hess, 2007): ‘When I'm out of the country for a while, I miss The Netherlands’, ‘I would regret having to move to another country’, ‘When I’ve been out of the country for a while, I’m happy to come back’ (1 = *strongly disagree*, 7 = *strongly agree*; α = .83).

#### Demographic characteristics

We controlled for sex (0 = *women*, 1 = *men*), age (in years), education level (1 = *primary education*, 8 = *doctorate*). We treated age and education as continuous variables.

### Data analytic strategy

We used confirmatory factor analysis in Mplus software (version 8.3; Muthén & Muthén, 1998–2017) to test whether the items measuring CPO, exclusive determination right, immigrant minority attitudes, national identification, and place attachment captured separate latent constructs. Next, we specified a structural equation model in which we regressed immigrant minority attitudes and European integration attitudes on CPO, mediated by exclusive determination right. We included control variables as predictors of the dependent variables and mediator. Finally, we added political ideology as a predictor of the dependent variables and as a moderator of the relationships among exclusive determination right, immigrant minority attitudes, and European integration attitudes. Further, we used ordinary least squares regression analysis with robust maximum likelihood estimation (MLR) to account for non‐normally distributed endogenous variables. We also used the full information maximum likelihood procedure (FIML), which allows missing values in endogenous variables, assuming missingness at random. We therefore endogenized exogenous variables with missing variables by estimating their variance. See Table 1 for the number of valid responses per variable.

**Table 1:** Descriptive statistics in Study 1

	Valid *n*	Range	Mean/proportion	*SD*	α	*t*	Correlations among main variables					
							2.	3.	4.	5.	6.	7.
1. Collective psychological ownership	572	1–7	4.78	1.65	.951	11.36***	.643***	–.318***	–.397***	.342***	.564***	.324***
2. Exclusive determination right	572	1–7	4.15	1.79	.963	2.01	1	–.407***	–.469***	.349***	.472***	.239***
3. Immigrant minority attitudes	566	1–11	6.13	1.65	.938	1.86		1	.505***	–.387***	–.263***	–.120*
4. European integration attitudes	564	1–7	3.69	1.78	–	4.14***			1	–.423***	–.293***	–.176**
5. Political ideology	572	1–5	2.75	1.17	–	5.13***				1	.279***	.126**
6. National identification	572	1–7	5.05	1.36	.874	18.56***					1	.710***
7. Place attachment	572	1–7	4.52	1.50	.831	8.23***						1
8. Sex (male)	570	0/1	0.59	–	–							
9. Education level		1–8	5.22	1.90	–							
10. Age	572	19–87	60.17	13.00	–							

Note

Descriptive statistics were based on manifest mean scores. Correlations were between latent factors and manifest single items. α is Cronbach's alpha*. t* is the *t*‐statistic of difference from the midpoint of the scale.

* *p *< .05; **p < .01; ***p < .001.

## Results and discussion

### Measurement model

The expected 5‐factor model did not fit the data well (CFI = .861, RMSEA = .099, SRMR = .064). Modification indices suggested that the factor for immigrant minority attitudes did not sufficiently account for variation in the 10 items. Thus, we specified five meaningful factors of two items each (Antilleans and Surinamese; Bulgarians and Poles; Moroccans and Turks; asylum seekers and refugees; people who entered the Netherlands illegally and people who overstayed their resident permits) and loaded them on a second‐order factor. This allowed us to account for the multidimensionality within the factor while using general immigrant minority attitudes as the dependent variable. A second‐order factor is a more parsimonious solution than specifying error covariances, and it reflects better the theoretically meaningful multidimensionality (Brown, 2015). We obtained a model fit (CFI = .960, RMSEA = .054, SRMR = .056) that was significantly better than the previous model and better than alternative 4‐factor solutions (Appendix S1). All items loaded significantly on their respective factor with loadings above .74.

### Descriptive statistics

Table 1 shows that participants held neutral attitudes towards immigrant minorities and thought that European integration had gone a bit too far. Further, they slightly agreed with the CPO items and the exclusive determination right items. CPO and exclusive determination right were positively related (*r* = .64). All correlations were significant and in the expected direction.

### Structural model

We regressed immigrant minority attitudes and European integration attitudes on CPO, mediated by exclusive determination right and including all control variables. The standardized total effects show that CPO was related to more negative attitudes towards immigrant minorities (β = −.244, *SE* = .052, *p *< .001) and European integration (β = −.341, *SE* = .052, *p *< .001), consistent with H1a and H1b (see all results, including all control variables, in Appendix S2). To compare the magnitude of these results, we also obtained standardized total effects of the main control variables. These show that CPO was a stronger predictor than national identification (β_immigrants_ = −.214, *SE* = .088, *p* = .014 and β_EU_ = −.119, *SE* = .083, *p* = .155) and place attachment (β_immigrants_ = .152, *SE* = .078, *p* = .051 and β_EU_ = .074, *SE* = .072, *p* = .304). Furthermore, indirect associations indicate that the association between CPO and immigrant minority attitudes was mediated by exclusive determination right (β = −.169, *SE* = .036, *p *< .001, 95% CI [−0.238, −0.099]^4^), in line with H2a. No direct relationship remained (β = −.076, *SE* = .059, *p* = .198). Consistent with H2b, the association between CPO and European integration attitudes was partially mediated by exclusive determination right (β = −.182, *SE* = .035, *p *< .001, 95% CI [−0.249, −0.115]), given that a direct negative path remained (β = −.159, *SE* = .061, *p* = .009).

**Figure 1:**
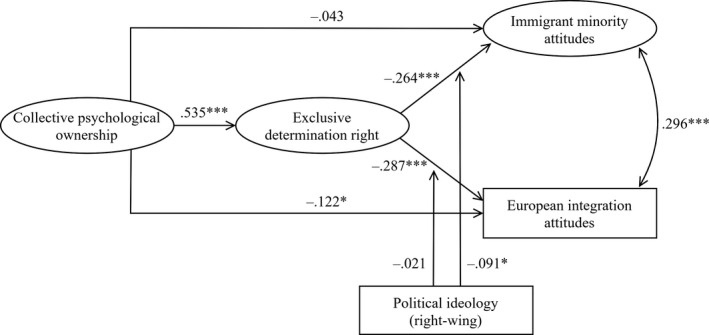
Standardized coefficients of the main paths of the final structural equation model in Study 1 (*N* = 572). Included control variables were not reported. *p < .05; ***p < .001.

**Figure 2:**
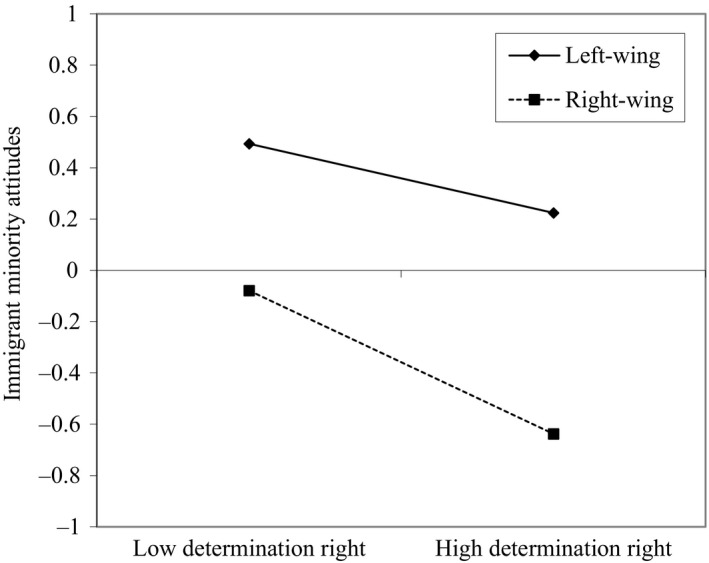
Simple slopes from the interaction between political ideology and exclusive determination right on immigrant minority attitudes in Study 1. Low determination right is 1 standard deviation below the mean of exclusive determination right (−1) and high determination right is 1 standard deviation above the mean (1). The *Y*‐axis represents the standardized scale of the latent dependent variable.

Figure 1 shows the standardized coefficients of the full model with interactions with political ideology. The negative relationship between exclusive determination right and immigrant minority attitudes was especially strong for right‐wing participants, as indicated by the negative interaction term (β = −.091, *SE* = .041, *p* = .026). The unstandardized simple slopes in Figure 2show that, for left‐wing participants (one standard deviation [*SD*] below the mean of political ideology), exclusive determination right was related to more negative immigrant minority attitudes (*b* = −.135, *SE* = .061, *p* = .026), but this association was stronger for right‐wing participants (1 *SD* above the mean of political ideology) (*b* = −.279, *SE* = .055, *p *< .001). This finding is consistent with H4. However, the relationship between exclusive determination right and European integration attitudes was not moderated by political ideology (β = −.021, *SE* = .037, *p* = .572).

## STUDY 2

We examined the cross‐national robustness of the findings by re‐testing the hypotheses that CPO is related to more negative attitudes towards immigrant minorities (H1a) and European integration (H1b) and that these associations are mediated by exclusive determination right (H2a and H2b). Additionally, we tested whether these negative attitudes towards immigrant minorities and European integration in turn accounted for pro‐Brexit voting (H3). We again considered the moderating influence of political ideology (H4). Moreover, we controlled for adherence to sovereignty along with national identification, place attachment, and demographic characteristics.

### Sample

We recruited a sample of native British adults via the Kantar Public (2019) online platform, which targeted a sample that would match the British population in terms of sex, age, education level, and country (England, Scotland, Wales). We collected these data between 25 February and 5 March 2019, approximately one month before the initial Brexit deadline. In total, 502 participants completed the survey.^5^ To test the main model with 21 observed indicators and three latent variables, we needed a minimum of 400 respondents for detecting a medium‐sized effect (Cohen's *d* = 0.4) at the .05 alpha level (Soper, 2017). We aimed for a larger sample size, because the final model that included control variables was more complex. We excluded seven participants who gave uniform answers to all questions, resulting in a final sample of 495 (249 [50%] women, 246 men), ranging in age from 18 to 84 years (*M* = 47.60, *SD* = 16.54). Of them, 11% had lower educational level (no education, primary school, or lower secondary education), 49% intermediate educational level (secondary education oriented towards vocational training or upper secondary education), and 40% higher educational level (tertiary higher professional education or university education). We added weights to correct for deviations from the targeted quotas, thereby making the findings representative for the British majority population.^6^

### Measures

#### Collective psychological ownership

Participants viewed the same instructions as in Study 1 and responded to four items: ‘We the Brits own this country’, ‘This country belongs to us Brits’, ‘I would agree with someone who says this country is collectively owned by us Brits’, ‘I feel as though we Brits own this country together’ (1 = *strongly disagree*, 7 = *strongly agree*; α = .96).

#### Exclusive determination right

We assessed this construct as in Study 1, only in reference to the British (α = .96).

#### Immigrant minority attitudes

We used the same feeling thermometer as in Study 1, but with 10 immigrant minority groups that are relevant in the British context: Caribbean Blacks, Bangladeshis, Indians, Pakistanis, Poles, Refugees, Romanians, Russians, Muslims, Turks (α = .97).

#### European integration attitudes

We measured this construct as in Study 1, except that we rephrased it in past tense (e.g. ‘European integration should have gone a lot further’), given that the electorate had already voted for Brexit.

#### Brexit voting

We assessed this construct with the question: ‘What did you vote in the 2016 Referendum?’ The options were as follows: *leave*, *remain*, *I chose not to vote*, *I was not allowed to vote*, and *prefer not to say*. We treated this variable as a dummy in which we coded ‘leave’ as 1 and ‘remain’ as 0. Four hundred and twenty‐one participants voted either ‘leave’ or ‘remain’, and we treated the responses of the remaining 74 participants on this variable as missing. We used this voting question in our main analyses as a proxy for voting behaviour, meaning that we predicted past voting behaviour with current attitudes. Therefore, in an alternative model, we ran the analyses with a different dependent variable, asking ‘if a second referendum were to be held today, what would you vote?’ We again treated this variable as a dummy in which we coded ‘leave’ as 1 and ‘remain’ as 0. Sixty‐six participants answered they would not vote or preferred not to answer and were treated as missing.

#### Political ideology

We measured this construct as in Study 1.

#### National identification

We measured this construct as in Study 1 (α = .91).

#### Place attachment

We used three items that were similar to the ones in Study 1: ‘I feel attached to Great Britain as a country’, ‘I would regret having to move to another country’, ‘Great Britain feels like my home’ (1 = *strongly disagree*, 7 = *strongly agree*; α = .85).

#### Adherence to sovereignty

We generated four items to assess this construct based on definitions of sovereignty: ‘International organizations should never interfere in national political decisions’, ‘National decision making should never be subject to international rules or regulations’, ‘An independent state should be free from external control’, ‘National decisions should be based on what the people want, instead of what international companies want’ (1 = *strongly disagree*, 7 = *strongly agree*; α = .86).

#### Demographic characteristics

We again controlled for sex (0 = *women*, 1 = *men*), age, and education (1 = *primary education not completed*, 14 = *PhD doctorate*). We also controlled for country of residence (England, Scotland, Wales), with England as the reference category.

### Data analytic strategy

We tested via confirmatory factor analysis whether the items measuring CPO, exclusive determination right, immigrant minority attitudes, national identification, place attachment, and adherence to sovereignty captured separate latent constructs. Subsequently, we specified a structural equation model of sequential mediation in which we regressed Brexit voting on CPO, mediated by (1) exclusive determination right and (2) immigrant minority attitudes and European integration attitudes. We included control variables as predictors of all endogenous variables. Next, we added political ideology as a predictor of immigrant minority attitudes and European integration attitudes, and as a moderator of the relationship between exclusive determination right and immigrant minority attitudes and European integration attitudes. We used logistic regression because of the dichotomous dependent variable Brexit voting and employed maximum likelihood estimation with a robust estimator (MLR) to account for non‐normally distributed endogenous variables. We opted for full information maximum likelihood (FIML), which allows missing values in endogenous variables, assuming missingness at random. So, we included cases with missing values on Brexit voting in the full model, but the missing value points were implied by the observed values of all other variables (Enders & Bandalos, 2001).^7^ Except for Brexit vote, no other variables had missing values.

## Results and discussion

### Measurement model

The expected 6‐factor model fitted the data well (CFI = .940, RMSEA = .058, SRMR = .041) and significantly better than several alternative 5‐factor solutions (see Appendix S4). All items loaded significantly on their respective factor with standardized loadings above .64. To reduce complexity of the final model,^8^ we treated multiple‐item control variables as manifest mean scores. A model with only CPO, exclusive determination right, and immigrant minority attitudes as latent factors fitted the data well (CFI = .941, RMSEA = .073, SRMR = .037).

### Descriptive statistics

Table 2 shows that participants held slightly negative immigrant minority attitudes and thought that European integration had gone a bit too far. Further, participants slightly agreed with both the CPO items and the exclusive determination right items. CPO and exclusive determination right were strongly positively related (*r* = .81).

**Table 2:** Descriptive statistics in Study 2

	Valid *n*	Range	Mean/ proportion	*SD*	α	*t*	Correlations among main variables							
							2.	3.	4.	5.	6.	7.	8.	9.
1. Collective psychological ownership	495	1–7	5.05	1.58	.958	14.71***	.810***	–.334***	–.328***	.311***	.257***	.589***	.559***	.486***
2. Exclusive determination right	495	1–7	5.16	1.56	.963	16.57***	1	–.312***	–.317***	.341***	.213***	.516***	.487***	.550***
3. Immigrant minority attitudes	495	1–11	5.34	2.13	.971	6.93***		1	.307***	–.340***	–.158**	–.118*	–.063	–.290***
4. European integration attitudes	495	1–7	2.80	1.59	–	16.81***			1	–.518***	–.297***	–.203***	–.151**	–.361***
5. Brexit vote	435	0/1	0.51	–	–					1	.296***	.211***	.170***	.401***
6. Political ideology	495	1–5	2.98	1.06	–	0.48					1	.219***	.172***	.232***
7. National identification	495	1–7	5.44	1.43	.910	22.40***						1	.760***	.309***
8. Place attachment	495	1–7	5.47	1.37	.847	23.97***							1	.320***
9. Adherence to sovereignty	495	1–7	5.07	1.20	.861	19.95***								1
10. Sex (male)	495	0/1	0.49	–	–									
11. Education level		1–14	8.15	3.57	–									
12. Age	495	18–84	46.05	17.09	–									
13. Country					–									
England	495	0/1	0.88	–	–									
Scotland	495	0/1	0.08	–	–									
Wales	495	0/1	0.04	–	–									

Note

Descriptive statistics were based on manifest mean scores. For the correlations, only CPO, exclusive determination right, and immigrant minority attitudes were treated as latent, as in the final model. α is Cronbach's alpha*. t* is the *t*‐statistic of difference from the midpoint of the scale. All statistics were based on the weighted data.

* *p *< .05; **p < .01; ***p < .001.

### Structural model

A sequential mediation model in which we regressed Brexit voting on CPO, mediated by exclusive determination right and subsequently by immigrant minority attitudes and European integration attitudes, suggested that the particularly high correlation between CPO and exclusive determination right led to multicollinearity issues. For example, whereas bivariate correlations of both CPO and exclusive determination right with European integration attitudes were significant (*r* = −.33 and *r* = −.32, respectively), they became non‐significant (β = −.221, *SE* = .108, *p* = .051 and β = −.146, *SE* = .102, *p* = .150 respectively) when simultaneously added as predictors of European integration attitudes, most likely as they cancelled each other out due to shared variance (see Appendix S5 for the full results). The large standard errors suggest multicollinearity (Grewal, Cote, & Baumgartner, 2004), making the results unreliable. We focused therefore only on CPO in our subsequent models (but see Footnote 10 for an alternative model with exclusive determination right as the sole predictor). Consequently, we did not test the mediation via exclusive determination right (H3a), but we did test moderation of the relationships among (1) CPO and immigrant minority attitudes, and (2) CPO and European integration attitudes, by political ideology.

We regressed Brexit voting on CPO, mediated by immigrant minority attitudes and European integration attitudes and including all control variables.^9^ The standardized results show that CPO was related to more negative attitudes towards immigrant minorities (β = −.344, *SE* = .065, *p *< .001) and the EU (β = −.214, *SE* = .068, *p* = .002), consistent with H1a and H1b as in Study 1 (see all results, including control variables, in Appendix S6). The standardized total effects also indicate that CPO was related to a higher likelihood of having voted ‘leave’ in the Brexit referendum (β = .194, *SE* = .075, *p* = .009), consistent with H3. This association was mediated by both attitudes towards immigrant minorities (β = .054, *SE* = .021, *p* = .010, 95% CI [0.010, 0.098]) and attitudes towards European integration (β = .092, *SE* = .032, *p* = .004, 95% CI [0.026, 0.159]), consistent with H3b and H3c. No direct relationship remained (β = .048 *SE* = .069, *p* = .487). Comparisons between standardized associations suggest that CPO was a stronger predictor of immigrant minority attitudes than national identification (β = .004, *SE* = .072, *p* = .958), place attachment (β = .195, *SE* = .069, *p* = .005), and adherence to sovereignty (β = −.157, *SE* = .057, *p* = .005). It was also a stronger predictor of European integration attitudes and Brexit vote than national identification (β_EU_ = −.081, *SE* = .079, *p* = .302 and β_Brexit_ = .062, *SE* = .084, *p* = .455) and place attachment (β_EU_ = .136, *SE* = .079, *p* = .083 and β_Brexit_ = −.133, *SE* = .086, *p* = .188). However, sovereignty was a stronger predictor than CPO of both European integration attitudes (β = −.245, *SE* = .061, *p *< .001) and Brexit vote (β = .361, *SE* = .068, *p *< .001).

**Figure 3:**
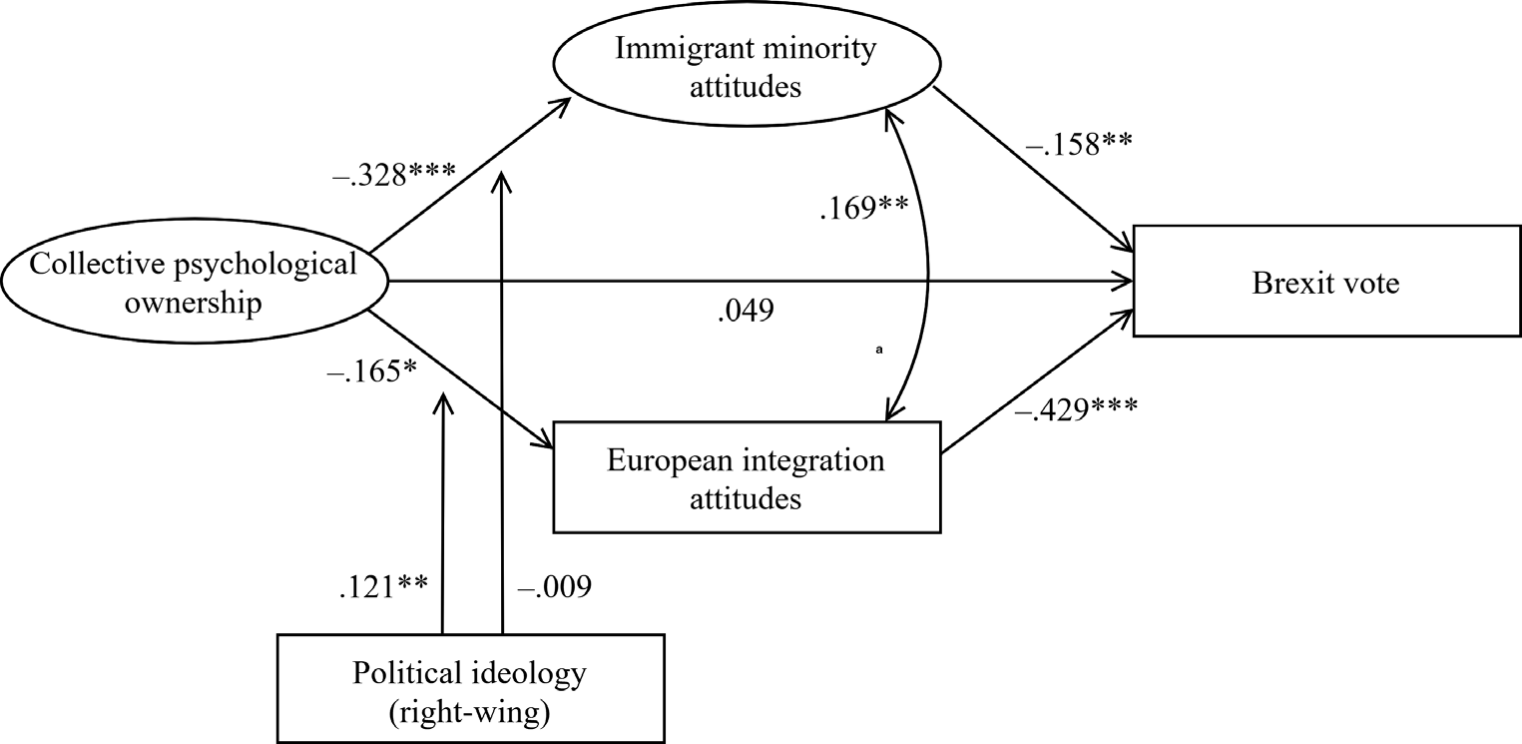
Standardized coefficients of the main paths of the final structural equation model in Study 2 (*N* = 495). Included control variables were not reported. *p < .05; **p < .01; ***p < .001.

**Figure 4:**
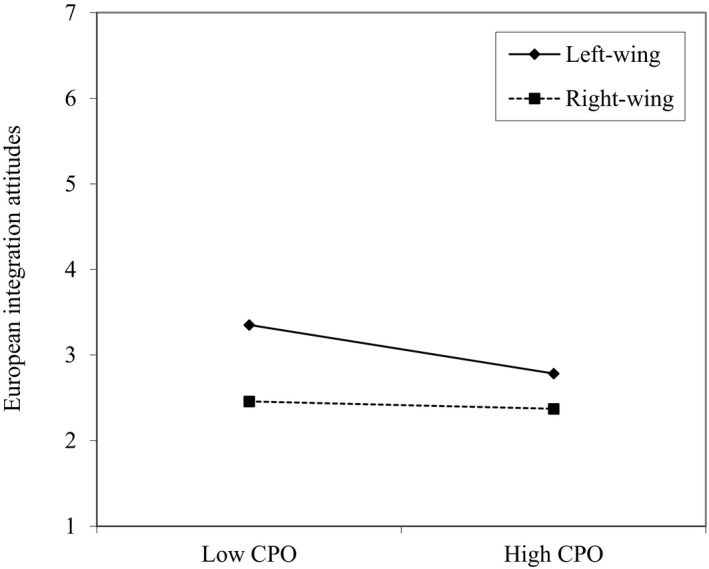
Simple slopes for the interaction between political ideology and CPO on European integration attitudes in Study 2. Low CPO is 1 Standard Deviation below the mean of CPO (−1) and high CPO is 1 Standard Deviation above the mean (1).

Figure 3 shows the standardized coefficients of the full model along with interactions with political ideology. We found no moderation of the relationship between CPO and immigrant minority attitudes (β = −.009, *SE* = .042, *p* = .831), in contrast to Study 1.^10^ Political ideology was also unrelated to immigrant minority attitudes, when the interaction was excluded (β = −.053, *SE* = .048, *p* = .273). Unexpectedly, the negative relationship between CPO and European integration attitudes was especially strong among left‐wing participants, as indicated by the positive interaction term (β = .121, *SE* = .039, *p* = .002). The unstandardized simple slopes in Figure 4show that, for left‐wing participants (1 *SD* below the mean of political ideology), CPO was significantly related to more negative European integration attitudes (*b* = −.285, *SE* = .080, *p *< .001), but that this association was less strong and non‐significant for right‐wing participants (1 *SD* above the mean of political ideology) (*b* = −.044, *SE* = .072, *p* = .543).

### Alternative model

In the original model, we predicted past voting behaviour on the basis of current attitudes. Participants, however, might have changed their attitudes or voting preferences. Therefore, we ran the analyses with an alternative dependent variable, asking what participants would vote today. The results did not differ substantially from the final model discussed (Appendix S8). This is unsurprising, given that only 7% of the ‘leave’ voters in the 2016 referendum indicated they would change their vote to ‘remain’ and 6% of the ‘remain’ voters would change their vote to ‘leave’.

### GENERAL DISCUSSION

Our research suggests that the right‐wing populist horizontal (natives vs. immigrants) and vertical (people vs. elite) ‘us‐them’ distinctions can be based not only on morality (the people are ‘good’; Mudde, 2007), but also on entitlement derived from ownership. Most people, for example, would endorse the notion that the owner of a house can decide who is welcome, and our findings suggest that people apply this logic to their country as a basis for their exclusionary attitudes and behaviours.

We demonstrated that *exclusive determination right* is in part responsible for the anti‐immigrant and anti‐EU attitudes and behaviours. We found high correlations between CPO and exclusive determination right in both studies, and indirect effects of CPO through exclusive determination right in Study 1. These findings are consistent with exclusive determination right being a core facet of ownership. The high correlations, especially in Study 2, raise the prospect of construct (in)distinguishability. However, although there will often be a close psychological connection between perceived ownership and exclusive determination right, ownership involves various other rights (right to use and transfer; Snare, 1972), and people with a sense of CPO may also grant others the right to (co‐)determination. Further, the association between CPO and determination rights differed between our two studies, and the stronger association in Study 2 is probably due to the specific national context. We collected the Study 2 data in the heat of the Brexit debate, in which ownership rhetoric and determination rights were highly prominent in the UK media.

We also examined whether those oriented to the political right were more likely to translate CPO to anti‐immigrant and anti‐EU attitudes, assuming that they have fewer problems with inequalities and value the status quo more than those oriented to the political left. In Study 1’s Dutch sample, gatekeeper right and exclusionary attitudes towards immigrant minorities were positively related for participants on both ends of the political spectrum, but, as expected, this relation was stronger among right‐wing people. However, the association between gatekeeper right and negative attitudes towards European integration was not conditional upon political ideology.

This pattern of findings was reversed among Study 2’s British participants. Right‐wing and left‐wing British were similarly likely to translate CPO into more negative attitudes towards immigrant minorities. This could be explained by the finding that political orientation was unrelated to immigrant minority attitudes, which is consistent with the result from focus groups that left‐ and right‐wing British do not have markedly different immigration stances (Leruth & Taylor‐Gooby, 2019). In contrast to this and to what we expected, specifically for left‐wing people, CPO was related to opposition to European integration and, in turn, to voting ‘leave’ in the referendum. Right‐wing people might already have been strongly opposed to European integration to the extent that their attitudes were not affected by individual differences in ownership beliefs. In contrast, left‐wing people might be more pro‐EU, but, if they happened to endorse CPO, they could turn against the EU. The relatively Eurosceptic attitudes among the British participants suggest such a ceiling effect. From the perspective of a populist right‐wing politician, then, right‐wing individuals may already be on board, whereas left‐wing individuals can be mobilized to agree with his or her anti‐EU agenda. The ‘leave’ camp in the Brexit debate – mostly driven by the United Kingdom Independence Party – might have adopted an effective strategy in using ownership rhetoric to win over the doubting left‐wing voters, crucial in a majority rule referendum. Along these lines, research has shown that left‐wing New Zealanders opposed more strongly pro‐bicultural policy when reading a political speech in which historical injustices were negated (vs. recognized), whereas right‐wing New‐Zealanders were not moved by such a speech (Sibley, Liu, Duckitt, & Khan, 2008). There might be other reasons why the role of political orientation was inconsistent across our studies. The meaning of the left‐right dimension can vary across countries (Huber & Inglehart, 1995) – although not as much in established liberal democracies such as the Netherlands and the United Kingdom (Caprara *et al*., 2017; Piurko, Schwartz, & Davidov, 2011). Further, attitudes towards specific issue that are based in underlying political orientations vary in their ideological relevance across space and time (Jost, 2006).

Notably, the importance of CPO in explaining exclusionary attitudes was robust across cultural contexts. Our research design did not allow for testing directly country differences, but British participants appeared to adhere more to CPO and especially the exclusive determination right than Dutch participants. This may be due to cultural and historical reasons, but it may also be due to the ownership‐fuelled ‘Leave’ campaign in the British media or the overrepresentation of higher educated Dutch participants. A study comparing representative samples from several countries, and using a longitudinal design, could provide more insight into country differences as well as temporal fluctuations of CPO.

Adherence to sovereignty was a strong predictor of European integration attitudes and the Brexit vote. A reason is that the sovereignty items triggered attitudes towards the EU and Brexit. The items explicitly mentioned ‘international organizations’ and ‘international rules or regulations’ and were presented after the questions about the EU and Brexit, thus being subject to order or framing effects. There is another reason. The idea that national governments should decide on what is good for society, which is a crucial feature of sovereignty, was perhaps not sufficiently emphasized in the sovereignty measures (Ripstein, 2017). Such an emphasis might have lessened the overlap between sovereignty and ownership.

Our work has several limitations. First, CPO can be a contributor to exclusionary attitudes, but can also be used to justify negative attitudes towards immigrants, the EU, and the Brexit vote (Crandall & Eshleman, 2003). Experimental work indicates that people can use intergroup threat to justify attitudes towards minorities (Bahns, 2016), and longitudinal research suggests that a reason voting behaviour influences attitudes is because voters are more likely to adjust their opinions to political messages they are most exposed to (Rooduijn, van der Brug, & de Lange, 2016). The ownership‐fuelled ‘Leave’ campaign, then, might have increased the probability of Brexit voters using CPO to justify their vote. Our cross‐sectional design prevents conclusions about the direction of influence, with bidirectionality being likely. Instead, our findings support a theoretically plausible and important direction of influence, which is also bolstered by experimental work. For example, experiments on the endowment effect and mere ownership effect have found that ownership causally affects the value attached to an object (Morewedge & Giblin, 2015). Although ownership of the country might be harder to manipulate, manipulating ownership rhetoric in a political speech could inform causality. Furthermore, longitudinal investigations could examine associations among these constructs across time.

Second, we do not suggest that socially and politically complex phenomena, such as the Brexit vote, can be explained primarily by CPO. Instead, we argue that CPO is one of several key factors that is likely to have assisted the ‘leave’ camp to win over the majority of votes. Our results indicate that CPO is a more critical predictor of the Brexit vote than national identification and place attachment, but not as critical as adherence to sovereignty. Future research should examine populist attitudes (Hobolt, 2016), intergroup threat (Van de Vyver, Leite, Abrams, & Palmer, 2018), and national nostalgia (Sedikides & Wildschut, 2019), along with their interaction with CPO.

Third, we focused on the exclusionary consequences of CPO. Follow‐up research could address its inclusionary side. A sense of ownership can be shared, which can strengthen the belief that a country belongs to ‘all of us’ and that we are collectively responsible for how it functions. CPO can be accompanied by a sense of responsibility (Verkuyten & Martinovic, 2017) cascading into positive intragroup consequences, such as willingness to be politically active, volunteer, and make a contribution to society more generally.

In closing, our findings indicated that CPO can help explain negative attitudes towards immigrants and the EU, due to CPO implying an exclusive determination right. These attitudes were translated into Brexit voting. CPO might have contributed to swaying the vote in favour of Brexit.

## Conflicts of interest

All authors declare no conflict of interest.

## Author contributions

Tom Nijs (Conceptualization; Data curation; Formal analysis; Investigation; Methodology; Writing – original draft). Borja Martinovic (Conceptualization; Data curation; Funding acquisition; Supervision; Writing – review & editing). Maykel Verkuyten (Conceptualization; Writing – review & editing) Constantine Sedikides (Conceptualization; Writing – review & editing).

## Supporting information


**Appendix S1.** Confirmatory factor analyses in Study 1 (*N* = 572).
**Appendix S2.** Standardized regression coefficients for the final model of Study 1.
**Appendix S3.** Standardized regression coefficients for the alternative model in which cases with missing values on Brexit voting were deleted in Study 2.
**Appendix S4.** Confirmatory factor analyses in Study 2 (*N* = 495).
**Appendix S5.** Standardized regression coefficients for the alternative model with CPO and Gatekeeper right included simultaneously in Study 2.
**Appendix S6.** Standardized regression coefficients for the final model of Study 2.
**Appendix S7.** Standardized regression coefficients for the alternative model with exclusive determination right as the main predictor, Study 2.
**Appendix S8.** Standardized regression coefficients for the alternative model with the alternative Brexit question, Study 2.

## Data Availability

The data and code will be made available on Open Science Framework when the paper gets accepted for publication.

## Footnotes

^1^ We examine individual and not group‐level perceptions of CPO or perceived in‐group norms about whether ‘we’ are the owners. Although individual perceptions might depend on perceived in‐group norms (Bennett, 2014), we focus on individual perceptions that we expect to be most relevant in explaining individual attitudes and behavioural intentions.

^2^ We did not consider Northern Ireland, because of its nuanced relationship with the United Kingdom and the debate about the EU border with Ireland (Coakley, 2007).

^3^ Given that political ideology is an exogenous variable used in a latent interaction model, we could not retain cases with missing values in the analysis.

^4^ We calculated all confidence intervals in both studies using bootstrapping with 1,000 iterations and using an ML estimator, which did not substantially change the results.

^5^ The dataset included another 516 participants who received a different version of the questionnaire. This version did not contain the measures pertaining to the current study.

^6^ These weights were unavailable for Study 1.

^7^ A model in which we deleted cases with missing values on Brexit voting yielded similar results (Appendix S3).

^8^ Model fitting with a categorical dependent variable regressed on several latent predictors posed numerical problems for Mplus.

^9^ We did not endogenize dichotomous control variables (i.e., sex, Scotland, Wales), because such practice would pose numerical problems for Mplus. Therefore, these variables were uncorrelated with the latent predictor CPO.

^10^ When we substituted CPO for exclusive determination right as the main predictor (Appendix S7), all the results, including the interactions with political orientation, were similar with the results of the final model we discuss in the main text.
