# Supplementary material for: ‘This country is OURS’: The exclusionary potential of collective psychological ownership
Source: Br J Soc Psychol. 2020 Jun 7;60(1):e12386. doi: 10.1111/bjso.12386 (PMC7818273; doi:10.1111/bjso.12386)
Supplement: Supplementary file 1 — Appendix S1. Confirmatory factor analyses in Study 1 (N = 572). Appendix S2. Standardized regression coefficients for the final model of Study 1. Appendix S3. Standardized regression coefficients for the alternative model in which cases with missing values on Brexit voting were deleted in Study 2. Appendix S4. Confirmatory factor analyses in Study 2 (N = 495). Appendix S5. Standardized regression coefficients for the alternative model with CPO and Gatekeeper right included simultaneously in Study 2. Appendix S6. Standardized regression coefficients for the final model of Study 2. Appendix S7. Standardized regression coefficients for the alternative model with exclusive determination right as the main predictor, Study 2. Appendix S8. Standardized regression coefficients for the alternative model with the alternative Brexit question, Study 2. [file BJSO-60-171-s001.docx]

**SUPPORTING INFORMATION**

‘This Country is OURS’: The Exclusionary Potential of Collective Psychological Ownership

**APPENDICES**

| **Appendix S1.** Confirmatory factor analyses in Study 1 (*N=*572) | | | | | |
| --- | --- | --- | --- | --- | --- |
| Model | χ² (*df*) | ∆χ² (∆*df*) | CFI | RMSEA | SRMR |
| A: 5 Factors: CPO, exclusive determination right, national identification, place attachment, immigrant minority attitudes | 1601.575 (242)*** |  | .861 | .099 | .064 |
| B: 5 Factors: including a second order factor of immigrant minority attitudes | 632.147 (237)*** | 496.388 (5)***^a^ | .960 | .054 | .056 |
| C: 4 Factors (including second order factor): CPO and exclusive determination right combined | 1939.553 (241)*** | 389.118 (4)***^b^ | .826 | .111 | .086 |
| D: 4 Factors (including second order factor): national identification and place attachment combined | 903.835 (241)*** | 288.509 (4)***^b^ | .932 | .069 | .067 |
| ^a^ Difference in Chi square test (∆χ²) is compared to model A; ^b^ Differences in Chi square tests (∆χ²) are compared to model B; *** *p* < .001. | | | | | |

| **Appendix S2. S**tandardized regression coefficients for the final model of Study 1 | | | | | | |
| --- | --- | --- | --- | --- | --- | --- |
|  | Model 1: Model Without Moderation | | | Model 2: Model With Moderation | | |
|  | Immigrant minority attitudes | European integration attitudes | Exclusive determination right | Immigrant minority attitudes | European integration attitudes | Exclusive determination right |
| Collective psychological ownership | –.076 (.059) | –.159 (.061)** | .535 (.049)*** | –.043 (.056) | –.122 (.057)* | .535 (.049)*** |
| Exclusive determination right | –.315 (.057)*** | –.340 (.056)*** |  | –.264 (.056)*** | –.287 (.055)*** |  |
|  |  |  |  |  |  |  |
| Political ideology |  |  |  | –.255 (.047)*** | –.276 (.039)*** |  |
| Political ideology * exclusive determination right |  |  |  | –.091 (.041)* | –.021 (.037) |  |
|  |  |  |  |  |  |  |
| National identification | –.139 (.086) | –.037 (.081) | .239 (.076)** | –.097 (.085) | .010 (.079) | .249 (.078)** |
| Place attachment | .144 (.076) | .033 (.071) | –.120 (.062) | .089 (.076) | .008 (.070) | –.129 (.064)* |
| Sex (male) | .145 (.042)** | .147 (.036)*** | .003 (.035) | .150 (.041)*** | .152 (.035)*** | .001 (.035) |
| Education level | .092 (.041)* | .165 (.038)*** | –.075 (.035)* | .084 (.040)* | .155 (.038)*** | –.068 (.035) |
| Age | .028 (.042) | –.009 (.034) | .028 (.034) | .013 (.040) | –.025 (.032) | .038 (.034) |
|  |  |  |  |  |  |  |
| R^2^ | .207 | .286 | .443 | .260 | .339 | .447 |
| N | 572 |  |  | 572 |  |  |
| * *p <* .05; ** *p <* .01; *** *p* < .001. | | | | | | |

| **Appendix S3.** Standardized regression coefficients for the alternative model in which cases with missing values on Brexit voting were deleted in Study 2 | | | | | | |
| --- | --- | --- | --- | --- | --- | --- |
|  | Model 1: Model Without Moderation | | | Model 2: Model with Moderation | | |
|  | Immigrant minority attitudes | European integration attitudes | Brexit vote | Immigrant minority attitudes | European integration attitudes | Brexit vote |
| Collective psychological ownership | –.322 (.067)*** | –.192 (.067)** | .048 (.070) | –.312 (.068)*** | –.147 (.065)* | .049 (.070) |
| Immigrant minority attitudes |  |  | –.155 (.054)** |  |  | –.156 (.054)** |
| European integration attitudes |  |  | –.429 (.058)*** |  |  | –.427 (.057)*** |
|  |  |  |  |  |  |  |
| Political ideology |  |  |  | –.072 (.050) | –.202 (.053)*** |  |
| Political ideology * CPO |  |  |  | –.015 (.044) | .116 (.041)** |  |
|  |  |  |  |  |  |  |
| National identification | .029 (.077) | –.117 (.079) | .028 (.076) | .042 (.078) | –.085 (.084) | .028 (.076) |
| Place attachment | .202 (.072)** | .134 (.078) | –.023 (.078) | .191 (.073)** | .118 (.079) | –.023 (.078) |
| Adherence to sovereignty | –.216 (.059)*** | –.269 (.065)*** | .233 (.070)** | –.211 (.058)*** | –.229 (.062)*** | .234 (.071)** |
| Sex (male) | .050 (.046) | –.005 (.051) | –.069 (.051) | .053 (.046) | –.011 (.049) | –.070 (.051) |
| Education level | .184 (.055)** | .068 (.059) | –.040 (.051) | .188 (.056)** | .073 (.056) | –.040 (.051) |
| Age | –.059 (.057) | –.023 (.053) | .084 (.060) | –.048 (.056) | –.017 (.052) | .085 (.060) |
| Country (ref = England) |  |  |  |  |  |  |
| Scotland | –.055 (.039) | .033 (.046) | –.139 (.059)* | –.058 (.039) | .047 (.045) | –.139 (.059)** |
| Wales | .032 (.037) | .048 (.031) | –.048 (.037) | .026 (.037) | .039 (.030) | –.048 (.037) |
|  |  |  |  |  |  |  |
| R^2^ | .234 | .189 | .520 | .242 | .221 | .516 |
| N | 435 |  |  | 435 |  |  |
| * *p <* .05; ** *p <* .01; *** *p* < .001. | | | | | | |

| **Appendix S4.** Confirmatory factor analyses in Study 2 (*N=*495). | | | | | |
| --- | --- | --- | --- | --- | --- |
| Model | χ² (*df*) | ∆χ² (∆*df*) | CFI | RMSEA | SRMR |
| A: 6 Factors: CPO, exclusive determination right, immigrant minority attitudes, national identification, place attachment, sovereignty | 887.767 (335)*** |  | .940 | .058 | .041 |
| B: 5 Factors: CPO and exclusive determination right combined | 1551.097 (340)*** | 532.029 (5)*** | .869 | .085 | .048 |
| C: 5 Factors: CPO and sovereignty combined | 1369.301 (340)*** | 244.112 (5)*** | .889 | .078 | .070 |
| D: 5 Factors: exclusive determination right and sovereignty combined | 1301.032 (340)*** | 223.229 (5)*** | .896 | .076 | .064 |
| E: 5 Factors: national identification and place attachment combined | 949.909 (340)*** | 43.780 (5)*** | .934 | .060 | .042 |
| Difference in Chi square test (∆χ²) is compared to model A; *** *p* < .001 | | | | | |

| **Appendix S5.** Standardized regression coefficients for the alternative model with CPO and gatekeeper right included simultaneously in Study 2 | | | | |
| --- | --- | --- | --- | --- |
|  | Exclusive determination right | Immigrant minority attitudes | European integration attitudes | Brexit vote |
| Collective psychological ownership | .809 (.031)*** | –.238 (.081)** | –.211 (.108) | .091 (.109) |
| Exclusive determination right |  | –.120 (.079) | –.146 (.102) | .177 (.107) |
| Immigrant minority attitudes |  |  |  | –.188 (.056)** |
| European integration attitudes |  |  |  | –.480 (.060)*** |
|  |  |  |  |  |
| R^2^ | .655 | .117 | .116 | .442 |
| N | 495 |  |  |  |
| * *p <* .05; ** *p <* .01; *** *p* < .001. | | | | |

| **Appendix S6.** Standardized regression coefficients for the final model of Study 2 | | | | | | |
| --- | --- | --- | --- | --- | --- | --- |
|  | Model 1: Model Without Moderation | | | Model 2: Model with Moderation | | |
|  | Immigrant minority attitudes | European integration attitudes | Brexit vote | Immigrant minority attitudes | European integration attitudes | Brexit vote |
| Collective psychological ownership | –.344 (.065)*** | –.214 (.068)** | .048 (.069) | –.328 (.067)*** | –.165 (.065)* | .049 (.070) |
| Immigrant minority attitudes |  |  | –.158 (.055)** |  |  | –.158 (.055)** |
| European integration attitudes |  |  | –.431 (.058)*** |  |  | –.429 (.058)*** |
|  |  |  |  |  |  |  |
| Political ideology |  |  |  | –.052 (.048) | –.207 (.049)*** |  |
| Political ideology * CPO |  |  |  | –.009 (.042) | .121 (.039)** |  |
|  |  |  |  |  |  |  |
| National identification | .004 (.072) | –.081 (.079) | .028 (.077) | .006 (.073) | –.060 (.082) | .028 (.077) |
| Place attachment | .195 (.069)** | .136 (.079) | –.023 (.078) | .186 (.070)** | .130 (.079) | –.024 (.079) |
| Adherence to sovereignty | –.157 (.057)** | –.245 (.061)*** | .231 (.070)** | –.160 (.055)** | –.208 (.057)*** | .232 (.070)** |
| Sex (male) | .049 (.044) | –.029 (.048) | –.070 (.051) | .050 (.044) | –.036 (.046) | –.071 (.051) |
| Education level | .123 (.049)* | .035 (.055) | –.041 (.052) | .122 (.049)* | .039 (.052) | –.041 (.052) |
| Age | –.105 (.055) | –.086 (.052) | .087 (.062) | –.100 (.055) | –.081 (.050) | .087 (.062) |
| Country (ref = England) |  |  |  |  |  |  |
| Scotland | –.061 (.038) | .018 (.044) | –.136 (.058)** | –.063 (.038) | .033 (.042) | –.137 (.058)** |
| Wales | .029 (.035) | .037 (.028) | –.046 (.034) | .025 (.035) | .028 (.027) | –.046 (.035) |
|  |  |  |  |  |  |  |
| R^2^ | .197 | .174 | .511 | .200 | .214 | .507 |
| N | 495 |  |  | 495 |  |  |
| * *p <* .05; ** *p <* .01; *** *p* < .001. | | | | | | |

| **Appendix S7.** Standardized regression coefficients for the alternative model with exclusive determination right as the main predictor, Study 2 | | | | | | |
| --- | --- | --- | --- | --- | --- | --- |
|  | Model 1: Model Without Moderation | | | Model 2: Model With Moderation | | |
|  | Immigrant minority attitudes | European integration attitudes | Brexit vote | Immigrant minority attitudes | European integration attitudes | Brexit vote |
| Exclusive determination right | –.271 (.064)*** | –.163 (.064)* | .093 (.073) | –.258 (.064)*** | –.145 (.062)* | .093 (.073) |
| Immigrant minority attitudes |  |  | –.147 (.055)** |  |  | –.148 (.056)** |
| European integration attitudes |  |  | –.426 (.059)*** |  |  | –.424 (.059)*** |
|  |  |  |  |  |  |  |
| Political ideology |  |  |  | –.075 (.049) | –.209 (.049)*** |  |
| Political ideology * exclusive determination right |  |  |  | –.007 (.040) | .112 (.037)** |  |
|  |  |  |  |  |  |  |
| National identification | –.033 (.074) | –.109 (.078) | .027 (.076) | –.025 (.076) | –.075 (.083) | .027 (.076) |
| Place attachment | .172 (.075)* | .119 (.085) | –.037 (.083) | .164 (.075)* | .110 (.085) | –.037 (.083) |
| Adherence to sovereignty | –.142 (.064)* | –.243 (.060)*** | .214 (.067)** | –.141 (.062)* | –.195 (.056)*** | .215 (.068)** |
| Sex (male) | .035 (.045) | –.038 (.049) | –.070 (.051) | .038 (.045) | –.042 (.046) | –.070 (.051) |
| Education level | .126 (.051)* | .033 (.056) | –.040 (.052) | .127 (.051)* | .033 (.053) | –.040 (.052) |
| Age | –.098 (.057) | –.083 (.053) | .091 (.063) | –.089 (.056) | –.082 (.051) | .091 (.063) |
| Country (ref = England) |  |  |  |  |  |  |
| Scotland | –.061 (.042) | .018 (.043) | –.136 (.059)* | –.062 (.041) | .030 (.042) | –.136 (.060)* |
| Wales | .042 (.036) | .045 (.028) | –.050 (.036) | .036 (.036) | .035 (.028) | –.050 (.036) |
|  |  |  |  |  |  |  |
| R^2^ | .168 | .163 | .511 | .173 | .205 | .508 |
| N | 495 |  |  | 495 |  |  |
| * *p <* .05; ** *p <* .01; *** *p* < .001. | | | | | | |

| **Appendix S8.** Standardized regression coefficients for the alternative model with the alternative Brexit question, Study 2 | | | | | | |
| --- | --- | --- | --- | --- | --- | --- |
|  | Model 1: Model Without Moderation | | | Model 2: Model With Moderation | | |
|  | Immigrant minority attitudes | European integration attitudes | Brexit vote | Immigrant minority attitudes | European integration attitudes | Brexit vote |
| Collective psychological ownership | –.335 (.065)*** | –.212 (.068)** | –.016 (.068) | –.318 (.068)*** | –.163 (.065)* | –.016 (.069) |
| Immigrant minority attitudes |  |  | –.126 (.057)* |  |  | –.126 (.058)* |
| European integration attitudes |  |  | –.441 (.060)*** |  |  | –.439 (.060)*** |
|  |  |  |  |  |  |  |
| Political ideology |  |  |  | –.050 (.048) | –.206 (.049)*** |  |
| Political ideology * CPO |  |  |  | –.010 (.042) | .121 (.039)** |  |
|  |  |  |  |  |  |  |
| National identification | –.002 (.072) | –.082 (.079) | .108 (.079) | –.001 (.073) | –.061 (.082) | .109 (.080) |
| Place attachment | .192 (.070)* | .136 (.079) | .011 (.080) | .182 (.071)* | .130 (.079) | .011 (.080) |
| Adherence to sovereignty | –.164 (.057)** | –.247 (.061)*** | .232 (.073)** | –.168 (.055)** | –.209 (.057)*** | .232 (.073)** |
| Sex (male) | .048 (.044) | –.029 (.048) | –.040 (.052) | .099 (.089) | –.036 (.046) | –.040 (.052) |
| Education level | .119 (.049)* | .034 (.055) | –.008 (.051) | .117 (.049)* | .038 (.052) | –.008 (.051) |
| Age | –.107 (.055) | –.086 (.052) | .118 (.058) | –.101 (.055) | –.081 (.050) | .119 (.058) |
| Country (ref = England) |  |  |  |  |  |  |
| Scotland | –.061 (.038) | .018 (.044) | –.091 (.053) | –.063 (.038) | .033 (.042) | –.091 (.054) |
| Wales | .029 (.035) | .037 (.028) | –.015 (.032) | .025 (.035) | .028 (.027) | –.016 (.032) |
|  |  |  |  |  |  |  |
| R^2^ | .197 | .175 | .504 | .200 | .214 | .500 |
| N | 495 |  |  | 495 |  |  |
| * *p <* .05; ** *p <* .01; *** *p* < .001. | | | | | | |
